# What are the public health implications of the life course perspective?

**DOI:** 10.1080/16549716.2019.1603491

**Published:** 2019-05-07

**Authors:** Jens Aagaard-Hansen, Shane A. Norris, Helle Terkildsen Maindal, Mark Hanson, Caroline Fall

**Affiliations:** a Health Promotion, Steno Diabetes Center Copenhagen, Gentofte, Denmark; b MRC Developmental Pathways for Health Research Unit, Department of Paediatrics, School of Clinical Medicine, Faculty of Health Sciences, University of the Witwatersrand, Johannesburg, South Africa; c Department of Public Health, Aarhus University, Aarhus, Denmark; d Institute of Developmental Sciences and NIHR Southampton Biomedical Research Centre, University of Southampton and University Hospital Southampton, Southampton, UK; e MRC Lifecourse Epidemiology Unit, Faculty of Medicine, University of Southampton, Southampton, UK

**Keywords:** Evidence, plasticity, policy, prioritization, inter-generational impact

## Abstract

During the past decades innovative research has shown that exposure to harmful events during pregnancy and early infancy (‘the first 1000 days’) has an impact on health at subsequent stages of the life course and even across generations. Recently it has been shown that even the pre-conception period is of outmost importance, and other scholars have made the case that the 1000 days should be extended to a period of 8000 days post-conception. The present contribution aims to bridge further the gap between research evidence and public health policy by applying a holistic ‘full-cycle’ perspective. Thus, a conceptual framework is suggested for guiding public health prioritization, including the variables of ‘impact on the next generation’, ‘plasticity’ and ‘available interventions with documented impact’. This framework could guide decision makers in selecting at which stages of the life course to invest (and not), and furthermore it points to some pertinent research priorities.

## Background

There is strong evidence that early life conceptualized as the ‘first 1000 days of the life course (including fetal life and the first two years of infancy) is essential for later health trajectories [1]. Bundy et al. make the case that the current focus on the first 1000 days of the life course should be expanded to a more holistic focus on the first 8000 days [2]. They recommend two high-return and low-cost intervention packages for the periods of school age and late adolescence to secure the gains of investment in the first 1000 days. In this paper, we further expand the discussion of public health prioritization by introducing three key criteria and applying a ‘full-cycle’ life course perspective.

Risks for a large number of public health problems, including non-communicable diseases (NCDs), accumulate not only throughout an individual’s life from the embryo stage onwards, but can also be passed from one generation to the next [3–6].

**Figure 1.**
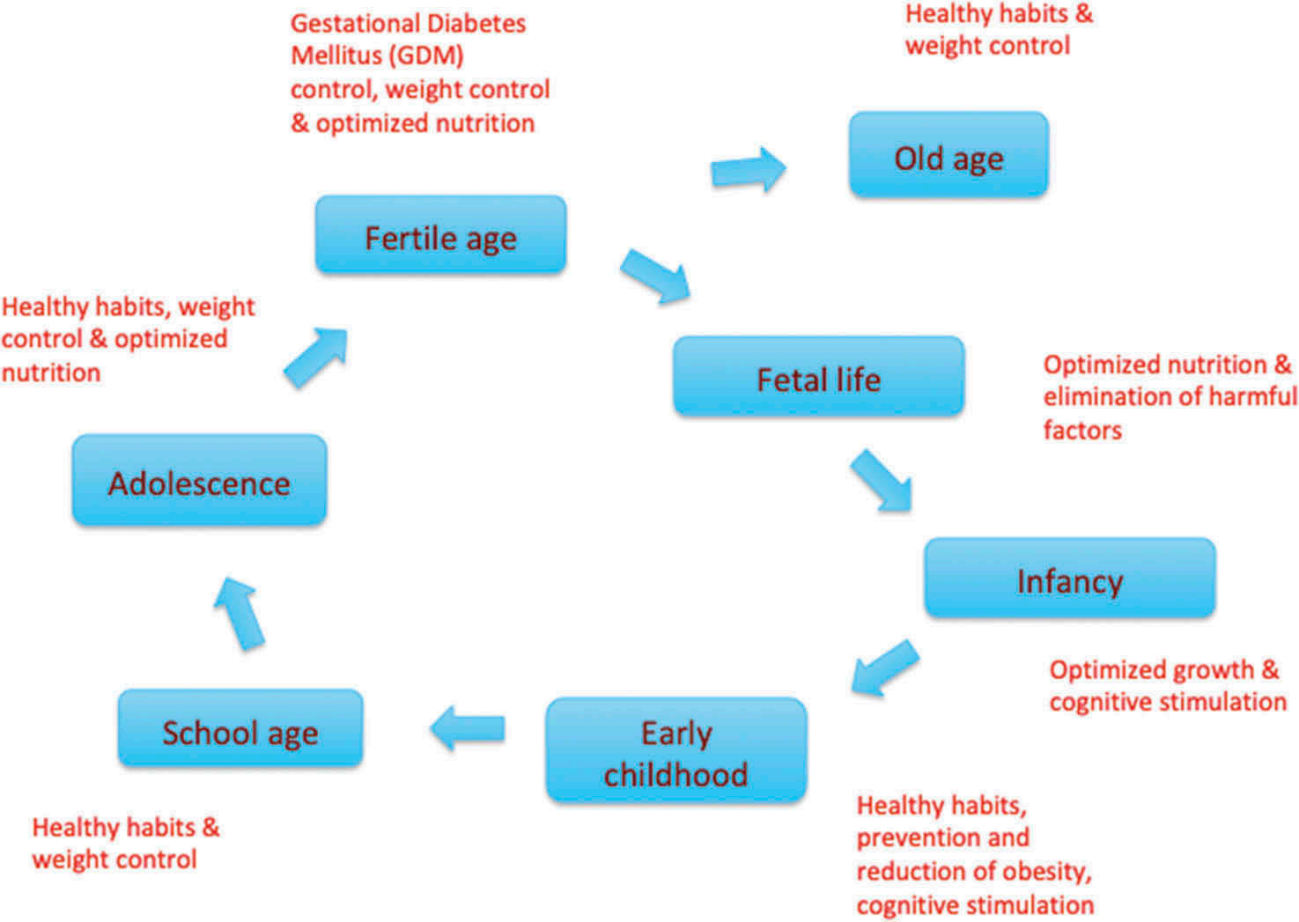
A circular display of the life course and examples of key priorities at various stages – the list is not exhaustive [1,2,7,10].

The life course concept can be visualized as a circle including the various stages of life: embryonic and fetal life, infancy, early childhood, school age, adolescence, and reproductive age (including pre-conception), where positive and negative events at any one stage may have an impact on subsequent stages and even across generations. Old age stands out as a tangent, where the impacts of events are not transmitted to the next generations (Figure 1).

## Public health implications

From a public health perspective interventions using this concept are attractive to the extent that they:

1. generate a ‘3 for the price of 1ʹ return on investment (better health for young people now, as future adults and for their children).
2. target stages in the life course where plasticity (an organism’s ability to adapt to its environment) is greatest;
3. have already been shown to be efficacious/effective (and cost-effective).

The two first criteria are both based on the notion that an investment in terms of a public health intervention should yield as much ‘value for money’ as possible; either by having impact on more than one condition or generation or by targeting persons when they are most receptive (i.e. have a high level of plasticity). It is more complicated to pinpoint the third criterion, as evidence varies according to the domain (Table 1). Bundy et al. presented the evidence for the impact on growth of interventions during an additional 7000 days beyond infancy [2]. Neuro-cognitive development is another area with solid evidence for effective interventions in early childhood on cognitive ability, educational outcome and lifetime earnings [7], whereas evidence for impact on NCDs is largely absent apart from long-term effects on mental health [8]. Factors influencing early life also have a bearing on the health and human capital of future generations [9]. There is mechanistic and observational evidence that the next generation can benefit from optimizing pregnant women’s health [10,11]. The rationale for pre-conception interventions is powerful, though not well-researched [12], and a recent review found no benefits on childhood health outcomes of maternal antenatal multiple micronutrient supplementation starting in mid-pregnancy [13]. Norris et al. have made an outline of an intergenerational cycle for priority in an African context [14].

**Table 1.** Assessment of impact on next generations, plasticity and availability of interventions with documented impact on growth, neuro-cognitive development and later development of NCDs at various stages of the life course as a guide to public health prioritizations.

Stage on life course	Impact on next generations	Plasticity	Available interventions with documented impact on growth	Available interventions with documented impact on neuro-cognitive development	Available interventions with documented impact on later development of NCDs
Fetal life	++	+++	+++	+++	-
Infancy	++	+++	+++	++	-
Early childhood	++	+++	+++	+	-
School age	++	++	++	-	-
Adolescence	+++	+	++	-	-
Fertile age (pre-conception and pregnancy)	+++	+	-	-	-
Old age	-	-	-	-	+

A life course perspective according to the criteria outlined in Table 1 provides a strong case for public health interventions during fetal life, early childhood, infancy [3,11], school age and adolescence [2], and measures to ensure optimal maternal body weight (neither under- nor overweight) on entering pregnancy [10]. Nevertheless, a substantial number of resources for prevention are dedicated to older stages of the life course where the advantages of intergenerational impact and plasticity are less likely to be reaped. This was most recently illustrated by a call for relatively greater attention to and prioritization of adolescent health and well-being [15,16]. Public health priorities should be based on ethical as well as rational, utilitarian considerations [17]. Thus, interventions during old age are still justified, though the impact may be less obvious or long-lasting than interventions at other stages of the life course. Interventions addressing specific stages of the life course should be planned together with a number of structural interventions, which work at the population level, e.g. targeted food taxes and subsidies or food labeling [18]. Combining these strategies not only addresses different generations, but also different socioeconomic groups, and is particularly pertinent in low- or middle-income countries.

## Conclusion

We contend that public health decision-makers have not fully embraced the consequences of the life course perspective. The analytical framework outlined above contributes to a systematic selection of interventions that provide most return on investment. This would lead to a shift of priorities to the parts of the life course ranging from the pre-conception period up to adolescence instead of, for instance, old age, and to domains where there is strong evidence for the effect of interventions such as on neuro-cognitive development. However, although much evidence has been accumulated over the past decades, more is needed. At an operational level, implementation measurement frameworks should be put in place in countries that already apply the life course perspective in their public health planning [6]. Furthermore, there is a pressing need to study the long-term impact of interventions during pre-conception and pre-natal periods as well as in infancy and early childhood, when plasticity and thus potential impact are highest. There are at least two challenges for future studies. These are (1) To design and sustain long-term studies in order to explore potential effects on e.g. NCD development (as causality chains often span more than one generation); (2) To broaden the scope of causal factors from the current dominant focus on nutrition to include the role of infections, pollutants, nurturing care, stress and unhealthy environments [12,19].

## Paper context

In recent decades a wealth of new evidence has emerged on accumulation of health risk along the life course and across generations, but translation to public health policy has been minimal. The present contribution aims to bridge this gap by setting up a conceptual framework for public health prioritization including ‘impact on next generation’, ‘plasticity’ and ‘interventions with documented impact’.
